# Supplementary material for: The paraventricular thalamus is a critical mediator of top-down control of cue-motivated behavior in rats
Source: eLife. 2019 Sep 10;8:e49041. doi: 10.7554/eLife.49041 (PMC6739869; doi:10.7554/eLife.49041)
Supplement: Supplementary file 1. — The results of linear mixed model analyses are shown for the effect of treatment (VEH vs. CNO) across sessions 1–5 of Pavlovian conditioned approach (PavCA) training for lever-directed behaviors, (lever contacts, probability to contact the lever and latency to contact the lever). Analyses were conducted separately for each experimental group (ST-Gq, GT-Gq, ST-Gi, GT-Gi, ST-no DREADD, GT-no DREADD). Bolded values indicate statistical significance, p<0.05. [file elife-49041-supp1.docx]

**Supplementary file 1. Acquisition of Pavlovian conditioned approach during PavCA Sessions 1-5: lever directed behaviors.**

|  | Lever-directed behaviors (Sign-tracking) | | | | | | | | | | |
| --- | --- | --- | --- | --- | --- | --- | --- | --- | --- | --- | --- |
|  | **ST-Gq** | | | | | | | | | | |
|  | Lever contacts | | |  | Probability lever | | |  | Latency lever | | |
|  | DF | F | p |  | DF | F | p |  | DF | F | p |
| Treatment | 1,22.854 | 2.347 | 0.139 |  | 1,25.783 | 4.368 | **<0.05** |  | 1,24.682 | 3.673 | 0.067 |
| Session | 4,32.488 | 29.080 | **<0.01** |  | 4,56.630 | 53.553 | **<0.01** |  | 4,59.141 | 49.482 | **<0.01** |
| Treatment*Session | 4,32.488 | 2.516 | 0.061 |  | 4,56.630 | 3.734 | **<0.01** |  | 4,59.141 | 3.875 | **<0.01** |
|  | **GT-Gq** | | | | | | | | | | |
|  | Lever contacts | | |  | Probability lever | | |  | Latency lever | | |
|  | DF | F | p |  | DF | F | p |  | DF | F | p |
| Treatment | 1,10.000 | 0.254 | 0.625 |  | 1,10.000 | 0.023 | 0.883 |  | 1,10.000 | 0.058 | 0.815 |
| Session | 4,10.000 | 3.717 | **<0.05** |  | 4,10.000 | 1.551 | 0.261 |  | 4,10.000 | 3.193 | 0.062 |
| Treatment*Session | 4,10.000 | 1.597 | 0.250 |  | 4,10.000 | 1.356 | 0.316 |  | 4,10.000 | 2.296 | 0.131 |
|  | **ST-Gi** | | | | | | | | | | |
|  | Lever contacts | | |  | Probability lever | | |  | Latency lever | | |
|  | DF | F | p |  | DF | F | p |  | DF | F | p |
| Treatment | 1,15.551 | 0.001 | 0.982 |  | 1,12.941 | 0.113 | 0.742 |  | 1,12.879 | 0.000 | 0.989 |
| Session | 4,46.408 | 8.800 | **<0.01** |  | 4,26.870 | 8.342 | **<0.01** |  | 4,33.398 | 16.017 | **<0.01** |
| Treatment*Session | 4,46.408 | 0.832 | 0.512 |  | 4,26.870 | 1.487 | 0.234 |  | 4,33.398 | 1.738 | 0.165 |
|  | **GT-Gi** | | | | | | | | | | |
|  | Lever contacts | | |  | Probability lever | | |  | Latency lever | | |
|  | DF | F | p |  | DF | F | p |  | DF | F | p |
| Treatment | 1,29.676 | 0.474 | 0.497 |  | 1,29.756 | 1.380 | 0.249 |  | 1,29.580 | 1.184 | 0.285 |
| Session | 4,29.339 | 2.842 | **<0.05** |  | 4,29.754 | 4.161 | **<0.05** |  | 4,66.666 | 1.612 | 0.182 |
| Treatment*Session | 4,29.339 | 0.388 | 0.816 |  | 4,29.754 | 0.061 | 0.993 |  | 4,66.666 | 1.205 | 0.317 |
|  | **ST-no DREADD** | | | | | | | | | | |
|  | Lever contacts | | |  | Probability lever | | |  | Latency lever | | |
|  | DF | F | p |  | DF | F | p |  | DF | F | p |
| Treatment | 1,19.424 | 0.065 | 0.801 |  | 1,16.672 | 0.200 | 0.661 |  | 1,16.238 | 0.079 | 0.782 |
| Session | 4,56.329 | 8.195 | **<0.01** |  | 4,46.156 | 18.797 | **<0.01** |  | 4,20.367 | 14,434 | **<0.01** |
| Treatment*Session | 4,56.239 | 0.638 | 0.638 |  | 4,46.156 | 0.840 | 0.507 |  | 4,20.367 | 1.100 | 0.383 |
|  | **GT-no DREADD** | | | | | | | | | | |
|  | Lever contacts | | |  | Probability lever | | |  | Latency lever | | |
|  | DF | F | p |  | DF | F | p |  | DF | F | p |
| Treatment | 1,13.133 | 4.440 | 0.055 |  | 1,17.211 | 3.680 | 0.072 |  | 1,18.443 | 3.223 | 0.089 |
| Session | 4,27.745 | 3.580 | **<0.05** |  | 4,45.408 | 3.928 | **<0.01** |  | 4,28.649 | 3.648 | **<0.05** |
| Treatment*Session | 4,27.745 | 2.663 | 0.053 |  | 4,45.408 | 2.865 | **<0.05** |  | 4,28.649 | 3.475 | **<0.05** |
