# Supplementary material for: The paraventricular thalamus is a critical mediator of top-down control of cue-motivated behavior in rats
Source: eLife. 2019 Sep 10;8:e49041. doi: 10.7554/eLife.49041 (PMC6739869; doi:10.7554/eLife.49041)
Supplement: Supplementary file 2. — The results of linear mixed model analyses are shown for the effect of treatment (VEH vs. CNO) across sessions 1–5 of Pavlovian conditioned approach (PavCA) training for magazine-directed behaviors (magazine entries, probability to enter the magazine, and latency to enter the magazine). Analyses were conducted separately for each experimental group (ST-Gq, GT-Gq, ST-Gi, GT-Gi, ST-no DREADD, GT-no DREADD). Bolded values indicate statistical significance, p<0.05. [file elife-49041-supp2.docx]

**Supplementary file 2. Acquisition of Pavlovian conditioned approach during PavCA Sessions 1-5: magazine-directed behaviors.**

|  | Magazine-directed behaviors (Goal-tracking) | | | | | | | | | | |
| --- | --- | --- | --- | --- | --- | --- | --- | --- | --- | --- | --- |
|  | **ST-Gq** | | | | | | | | | | |
|  | Magazine entries | | |  | Probability magazine | | |  | Latency magazine | | |
|  | DF | F | p |  | DF | F | p |  | DF | F | p |
| Treatment | 1,23.121 | 3.333 | 0.081 |  | 1,26.890 | 3.097 | 0.090 |  | 1,26.238 | 3.305 | 0.081 |
| Session | 4,22.146 | 12.299 | **<0.05** |  | 4,40.812 | 13.489 | **<0.01** |  | 4,42.943 | 10.076 | **<0.01** |
| Treatment*Session | 4,22.146 | 1.968 | 0.135 |  | 4,40.812 | 2.993 | **<0.05** |  | 4,42.943 | 2.013 | 0.110 |
|  | **GT-Gq** | | | | | | | | | | |
|  | Magazine entries | | |  | Probability magazine | | |  | Latency magazine | | |
|  | DF | F | p |  | DF | F | p |  | DF | F | p |
| Treatment | 1,10.820 | 0.744 | 0.407 |  | 1,9.529 | 0.180 | 0.681 |  | 1,10.516 | 0.150 | 0.706 |
| Session | 4,11.690 | 18.267 | **<0.01** |  | 4,22.183 | 29.082 | **<0.01** |  | 4,15.998 | 31.383 | **<0.01** |
| Treatment*Session | 4,11.690 | 0.495 | 0.740 |  | 4,22.183 | 0.210 | 0.930 |  | 4,15.998 | 0.238 | 0.913 |
|  | **ST-Gi** | | | | | | | | | | |
|  | Magazine entries | | |  | Probability magazine | | |  | Latency magazine | | |
|  | DF | F | p |  | DF | F | p |  | DF | F | p |
| Treatment | 1,12.252 | 0.363 | 0.558 |  | 1,14.596 | 0.202 | 0.660 |  | 1,14.197 | 0.729 | 0.407 |
| Session | 4,14.652 | 3.461 | **<0.05** |  | 4,25.325 | 5.141 | **<0.01** |  | 4,21.601 | 5.225 | **<0.01** |
| Treatment*Session | 4,14.652 | 0.765 | 0.565 |  | 4,25.325 | 0.759 | 0.561 |  | 4,21.601 | 0.751 | 0.568 |
|  | **GT-Gi** | | | | | | | | | | |
|  | Magazine entries | | |  | Probability magazine | | |  | Latency magazine | | |
|  | DF | F | p |  | DF | F | p |  | DF | F | p |
| Treatment | 1,29.689 | 0.015 | 0.902 |  | 1,31.280 | 0.300 | 0.588 |  | 1,31.044 | 0.155 | 0.697 |
| Session | 4,39.931 | 21.667 | **<0.01** |  | 4,52.002 | 31.364 | **<0.01** |  | 4,47.028 | 30.693 | **<0.01** |
| Treatment*Session | 4,39.931 | 0.296 | 0.879 |  | 4,52.002 | 0.342 | 0.849 |  | 4,47.028 | 0.937 | 0.937 |
|  | **ST-no DREADD** | | | | | | | | | | |
|  | Magazine entries | | |  | Probability magazine | | |  | Latency magazine | | |
|  | DF | F | p |  | DF | F | p |  | DF | F | p |
| Treatment | 1,18.485 | 0.042 | 0.840 |  | 1,18.503 | 0.903 | 0.354 |  | 1,20.538 | 1.087 | 0.309 |
| Session | 4,32.474 | 2.128 | 0.100 |  | 4,35.919 | 2.612 | 0.051 |  | 4,35.722 | 3.220 | **<0.05** |
| Treatment*Session | 4,32.474 | 1.111 | 0.368 |  | 4,35.919 | 0.443 | 0.777 |  | 4,35.722 | 0.447 | 0.773 |
|  | **GT-no DREADD** | | | | | | | | | | |
|  | Magazine entries | | |  | Probability magazine | | |  | Latency magazine | | |
|  | DF | F | p |  | DF | F | p |  | DF | F | p |
| Treatment | 1,14.876 | 0.748 | 0.401 |  | 1,15.127 | 0.094 | 0.763 |  | 1,15.061 | 0.078 | 0.784 |
| Session | 4,24.330 | 14.599 | **<0.01** |  | 4,31.623 | 14.955 | **<0.01** |  | 4,23.690 | 12.976 | **<0.01** |
| Treatment*Session | 4,24.330 | 1.538 | 0.223 |  | 4,31.623 | 2.040 | 0.113 |  | 4,23.690 | 1.730 | 0.177 |
