# Supplementary material for: The paraventricular thalamus is a critical mediator of top-down control of cue-motivated behavior in rats
Source: eLife. 2019 Sep 10;8:e49041. doi: 10.7554/eLife.49041 (PMC6739869; doi:10.7554/eLife.49041)
Supplement: Supplementary file 3. — The results of linear mixed model analyses are shown for the effect of treatment (VEH vs. CNO), session (rescreening vs, test) and treatment x session interaction for lever-directed behaviors (lever contacts, probability to contact the lever and latency to contact the lever). Analyses were conducted separately for each experimental group (ST-Gq, GT-Gq, ST-Gi, GT-Gi, ST-no DREADD, GT-no DREADD). Bolded values indicate statistical significance, p<0.05. [file elife-49041-supp3.docx]

**Supplementary file 3. PavCA rescreening (Sessions 6-10) vs. PavCA test (Sessions 11-16): lever-directed behaviors.**

|  | Lever-directed behaviors (Sign-tracking) | | | | | | | | | | |
| --- | --- | --- | --- | --- | --- | --- | --- | --- | --- | --- | --- |
|  | **ST-Gq** | | | | | | | | | | |
|  | Lever contacts | | |  | Probability lever | | |  | Latency lever | | |
|  | DF | F | p |  | DF | F | p |  | DF | F | p |
| Treatment | 1,23 | 8.533 | **<0.05** |  | 1,23 | 21.366 | **<0.01** |  | 1,23 | 12.327 | **<0.05** |
| Session | 1,23 | 7.884 | **<0.05** |  | 1,23 | 11.196 | **<0.05** |  | 1,23 | 17.735 | **<0.01** |
| Treatment*Session | 1,23 | 3.072 | 0.093 |  | 1,23 | 9.214 | **<0.05** |  | 1,23 | 6.526 | **<0.05** |
|  | **GT-Gq** | | | | | | | | | | |
|  | Lever contacts | | |  | Probability lever | | |  | Latency lever | | |
|  | DF | F | p |  | DF | F | p |  | DF | F | p |
| Treatment | 1,10 | 0.415 | 0.534 |  | 1,10 | 0.261 | 0.621 |  | 1,10 | 0.164 | 0.694 |
| Session | 1,10 | 0.081 | 0.782 |  | 1,10 | 0.062 | 0.808 |  | 1,10 | 0.508 | 0.492 |
| Treatment*Session | 1,10 | 0.014 | 0.908 |  | 1,10 | 0.008 | 0.929 |  | 1,10 | 0.003 | 0.957 |
|  | **ST-Gi** | | | | | | | | | | |
|  | Lever contacts | | |  | Probability lever | | |  | Latency lever | | |
|  | DF | F | p |  | DF | F | p |  | DF | F | p |
| Treatment | 1,12 | 0.683 | 0.425 |  | 1,12 | 0.135 | 0.720 |  | 1,12 | 0.389 | 0.545 |
| Session | 1,12 | 1.651 | 0.223 |  | 1,12 | 0.441 | 0.519 |  | 1,12 | 2.553 | 0.136 |
| Treatment*Session | 1,12 | 0.622 | 0.446 |  | 1,12 | 0.028 | 0.871 |  | 1,12 | 0.471 | 0.505 |
|  | **GT-Gi** | | | | | | | | | | |
|  | Lever contacts | | |  | Probability lever | | |  | Latency lever | | |
|  | DF | F | p |  | DF | F | p |  | DF | F | p |
| Treatment | 1,30 | 1.597 | 0.216 |  | 1,30 | 1.350 | 0.254 |  | 1,30 | 2.160 | 0.152 |
| Session | 1,30 | 4.595 | **<0.05** |  | 1,30 | 5.164 | **<0.05** |  | 1,30 | 4.673 | **<0.05** |
| Treatment*Session | 1,30 | 2.870 | 0.101 |  | 1,30 | 2.918 | 0.098 |  | 1,30 | 2.333 | 0.137 |
|  | **ST-no DREADD** | | | | | | | | | | |
|  | Lever contacts | | |  | Probability lever | | |  | Latency lever | | |
|  | DF | F | p |  | DF | F | p |  | DF | F | p |
| Treatment | 1,15 | 1.322 | 0.268 |  | 1,15 | 0.089 | 0.769 |  | 1,15 | 0.014 | 0.909 |
| Session | 1,15 | 18.467 | **<0.05** |  | 1,15 | 4.797 | **<0.05** |  | 1,15 | 16.326 | **<0.05** |
| Treatment*Session | 1,15 | 1.575 | 0.229 |  | 1,15 | 1.233 | 0.284 |  | 1,15 | 1.079 | 0.315 |
|  | **GT-no DREADD** | | | | | | | | | | |
|  | Lever contacts | | |  | Probability lever | | |  | Latency lever | | |
|  | DF | F | p |  | DF | F | p |  | DF | F | p |
| Treatment | 1,13 | 0.040 | 0.845 |  | 1,13 | 0.001 | 0.974 |  | 1,13 | 0.014 | 0.908 |
| Session | 1,13 | 1.663 | 0.220 |  | 1,13 | 1.927 | 0.188 |  | 1,13 | 2.977 | 0.108 |
| Treatment*Session | 1,13 | 0.602 | 0.452 |  | 1,13 | 0.265 | 0.616 |  | 1,13 | 0.000 | 0.992 |
