# Supplementary material for: The paraventricular thalamus is a critical mediator of top-down control of cue-motivated behavior in rats
Source: eLife. 2019 Sep 10;8:e49041. doi: 10.7554/eLife.49041 (PMC6739869; doi:10.7554/eLife.49041)
Supplement: Supplementary file 4. — The results of linear mixed model analyses are shown for the effect of treatment (VEH vs. CNO), session (rescreening vs, test) and treatment x session interaction for magazine-directed behaviors (magazine entries, probability to enter the magazine and latency to enter the magazine). Analyses were conducted separately for each experimental group (ST-Gq, GT-Gq, ST-Gi, GT-Gi, ST-no DREADD, GT-no DREADD). Bolded values indicate statistical significance, p<0.05. [file elife-49041-supp4.docx]

**Supplementary file 4. PavCA rescreening (Sessions 6-10) vs. PavCA test (Sessions 11-16): magazine-directed behaviors.**

|  | Magazine-directed behaviors (Goal-tracking) | | | | | | | | | | |
| --- | --- | --- | --- | --- | --- | --- | --- | --- | --- | --- | --- |
|  | **ST-Gq** | | | | | | | | | | |
|  | Magazine entries | | |  | Probability magazine | | |  | Latency magazine | | |
|  | DF | F | p |  | DF | F | p |  | DF | F | p |
| Treatment | 1,23 | 6.277 | **<0.05** |  | 1,23 | 6.674 | **<0.05** |  | 1,23 | 8.696 | **<0.05** |
| Session | 1,23 | 0.056 | 0.815 |  | 1,23 | 0.222 | 0.642 |  | 1,23 | 0.060 | 0.808 |
| Treatment*Session | 1,23 | 3.518 | 0.073 |  | 1,23 | 5.323 | **<0.05** |  | 1,23 | 5.947 | **<0.05** |
|  | **GT-Gq** | | | | | | | | | | |
|  | Magazine entries | | |  | Probability magazine | | |  | Latency magazine | | |
|  | DF | F | p |  | DF | F | p |  | DF | F | p |
| Treatment | 1,10 | 0.574 | 0.466 |  | 1,10 | 0.001 | 0.982 |  | 1,10 | 0.064 | 0.805 |
| Session | 1,10 | 0.078 | 0.786 |  | 1,10 | 0.003 | 0.957 |  | 1,10 | 1.815 | 0.208 |
| Treatment*Session | 1,10 | 0.460 | 0.513 |  | 1,10 | 0.609 | 0.453 |  | 1,10 | 0.009 | 0.924 |
|  | **ST-Gi** | | | | | | | | | | |
|  | Magazine entries | | |  | Probability magazine | | |  | Latency magazine | | |
|  | DF | F | p |  | DF | F | p |  | DF | F | p |
| Treatment | 1,12 | 1.116 | 0.312 |  | 1,12 | 0.439 | 0.520 |  | 1,12 | 0.046 | 0.834 |
| Session | 1,12 | 6.038 | **<0.05** |  | 1,12 | 7.279 | **<0.05** |  | 1,12 | 5.538 | **<0.05** |
| Treatment*Session | 1,12 | 2.502 | 0.140 |  | 1,12 | 2.462 | 0.143 |  | 1,12 | 2.184 | 0.165 |
|  | **GT-Gi** | | | | | | | | | | |
|  | Magazine entries | | |  | Probability magazine | | |  | Latency magazine | | |
|  | DF | F | p |  | DF | F | p |  | DF | F | p |
| Treatment | 1,30 | 0.459 | 0.503 |  | 1,30 | 3.271 | 0.081 |  | 1,30 | 3.416 | 0.074 |
| Session | 1,30 | 1.103 | 0.302 |  | 1,30 | 1.124 | 0.298 |  | 1,30 | 5.187 | **<0.05** |
| Treatment*Session | 1,30 | 1.842 | 0.185 |  | 1,30 | 1.801 | 0.190 |  | 1,30 | 2.761 | 0.107 |
|  | **ST-no DREADD** | | | | | | | | | | |
|  | Magazine entries | | |  | Probability magazine | | |  | Latency magazine | | |
|  | DF | F | p |  | DF | F | p |  | DF | F | p |
| Treatment | 1,15 | 0.352 | 0.562 |  | 1,15 | 0.430 | 0.522 |  | 1,15 | 0.347 | 0.564 |
| Session | 1,15 | 2.447 | 0.139 |  | 1,15 | 2.851 | 0.112 |  | 1,15 | 0.784 | 0.390 |
| Treatment*Session | 1,15 | 1.356 | 0.262 |  | 1,15 | 1.211 | 0.289 |  | 1,15 | 0.482 | 0.498 |
|  | **GT-no DREADD** | | | | | | | | | | |
|  | Magazine entries | | |  | Probability magazine | | |  | Latency magazine | | |
|  | DF | F | p |  | DF | F | p |  | DF | F | p |
| Treatment | 1,13 | 0.490 | 0.496 |  | 1,13 | 0.373 | 0.552 |  | 1,13 | 0.237 | 0.634 |
| Session | 1,13 | 0.198 | 0.664 |  | 1,13 | 0.025 | 0.876 |  | 1,13 | 0.428 | 0.525 |
| Treatment*Session | 1,13 | 0.101 | 0.756 |  | 1,13 | 0.060 | 0.810 |  | 1,13 | 0.000 | 0.986 |
