# Supplementary material for: The paraventricular thalamus is a critical mediator of top-down control of cue-motivated behavior in rats
Source: eLife. 2019 Sep 10;8:e49041. doi: 10.7554/eLife.49041 (PMC6739869; doi:10.7554/eLife.49041)
Supplement: Supplementary file 5. — The results of linear mixed model analyses are shown for the effect of treatment (VEH vs. CNO) across sessions 1–3 of Pavlovian conditioned approach (PavCA) training for lever-directed behaviors (lever contacts, probability to contact the lever and latency to contact the lever). Analyses were conducted separately for each experimental group (ST-Gq, GT-Gi). Bolded values indicate statistical significance, p<0.05. [file elife-49041-supp5.docx]

**Supplementary file 5. Acquisition of sign-tracking behavior during PavCA Sessions 1-3: lever-directed behaviors.**

|  | Lever-directed behaviors (Sign-tracking) | | | | | | | | |
| --- | --- | --- | --- | --- | --- | --- | --- | --- | --- |
|  | **ST-Gq** | | | | | | | | |
|  | Lever contacts | | | Probability lever | | | Latency lever | | |
|  | DF | F | p | DF | F | p | DF | F | p |
| Treatment | 1,12.377 | 4.080 | 0.066 | 1,12,137 | 0.411 | 0.411 | 1,12.402 | 1.481 | 0.246 |
| Session | 2,13.246 | 17.459 | **<0.05** | 2,15.117 | 29.102 | **<0.01** | 2,13.358 | 23.444 | **<0.01** |
| Treatment*Session | 2,13.246 | 0.014 | 0.986 | 2,15.117 | 0.179 | 0.838 | 2,13.358 | 0.636 | 0.545 |
|  | **GT-Gi** | | | | | | | | |
|  | Lever contacts | | | Probability lever | | | Latency lever | | |
|  | DF | F | p | DF | F | p | DF | F | p |
| Treatment | 1,11.728 | 4.607 | 0.054 | 1,8.748 | 5.054 | 0.052 | 1,10.028 | 5.116 | **<0.05** |
| Session | 2,18.000 | 3.360 | **<0.05** | 2,16.264 | 0.655 | 0.533 | 2,16.333 | 1.715 | 0.211 |
| Treatment*Session | 2,18.000 | 0.957 | 0.403 | 2,16.264 | 0.143 | 0.868 | 2,16.333 | 0.724 | 0.500 |
