# Supplementary material for: The paraventricular thalamus is a critical mediator of top-down control of cue-motivated behavior in rats
Source: eLife. 2019 Sep 10;8:e49041. doi: 10.7554/eLife.49041 (PMC6739869; doi:10.7554/eLife.49041)
Supplement: Supplementary file 6. — The results of linear mixed model analyses are shown for the effect of treatment (VEH vs. CNO) across sessions 1–3 of Pavlovian conditioned approach (PavCA) training for magazine-directed behaviors (magazine entries, probability to enter the magazine and latency to enter the magazine). Analyses were conducted separately for each experimental group (ST-Gq, GT-Gi). Bolded values indicate statistical significance, p<0.05. [file elife-49041-supp6.docx]

**Supplementary file 6. Acquisition of sign-tracking behavior during PavCA Sessions 1-3: magazine-directed behaviors.**

|  | Magazine-directed behaviors (Goal-tracking) | | | | | | | | |
| --- | --- | --- | --- | --- | --- | --- | --- | --- | --- |
|  | ST-Gq | | | | | | | | |
|  | Magazine entries | | | Probability magazine | | | Latency magazine | | |
|  | DF | F | p | DF | F | p | DF | F | p |
| Treatment | 1,12.090 | 1.382 | 0.262 | 1,13.564 | 1.162 | 0.300 | 1,13.282 | 1.171 | 0.299 |
| Session | 2,13.611 | 4.155 | **<0.05** | 2,24.414 | 1.696 | 0.204 | 2,24.046 | 2.422 | 0.110 |
| Treatment*Session | 2,13.611 | 0.480 | 0.629 | 2,24.414 | 0.523 | 0.599 | 2,24.046 | 0.697 | 0.508 |
|  | GT-Gi | | | | | | | | |
|  | Magazine entries | | | Probability magazine | | | Latency magazine | | |
|  | DF | F | p | DF | F | p | DF | F | p |
| Treatment | 1,10.277 | 3.810 | 0.079 | 1,9.381 | 4.416 | 0.064 | 1,9.460 | 4.983 | 0.051 |
| Session | 2,18.000 | 9.671 | **<0.05** | 2,12.030 | 16.367 | **<0.01** | 2,11.614 | 15.136 | **<0.05** |
| Treatment*Session | 2,18.000 | 2.501 | 0.110 | 2,12.030 | 1.832 | 0.202 | 2,11.614 | 1.533 | 0.256 |
