# Supplementary material for: The paraventricular thalamus is a critical mediator of top-down control of cue-motivated behavior in rats
Source: eLife. 2019 Sep 10;8:e49041. doi: 10.7554/eLife.49041 (PMC6739869; doi:10.7554/eLife.49041)
Supplement: Supplementary file 7. — The results of linear mixed model analyses are shown for the effect of treatment (VEH vs. CNO), session (3 vs. 6) and treatment x session interaction for lever-directed behaviors (lever contacts, probability to contact the lever and latency to contact the lever). Analyses were conducted separately for each experimental group (ST-Gq, GT-Gi). Bolded values indicate statistical significance, p<0.05. [file elife-49041-supp7.docx]

**Supplementary file 7. Session 3 vs. Session 6 of PavCA training: lever-directed behaviors.**

|  | Lever-directed behaviors (Sign-tracking) | | | | | | | | |
| --- | --- | --- | --- | --- | --- | --- | --- | --- | --- |
|  | ST-Gq | | | | | | | | |
|  | Lever contacts | | | Probability lever | | | Latency lever | | |
|  | DF | F | p | DF | F | p | DF | F | p |
| Treatment | 1,12 | 0.028 | 0.870 | 1,12 | 0.142 | 0.713 | 1,12 | 0.039 | 0.847 |
| Session | 1,12 | 0.244 | 0.630 | 1,12 | 0.163 | 0.694 | 1,12 | 0.164 | 0.692 |
| Treatment*Session | 1,12 | 2.244 | 0.160 | 1,12 | 0.007 | 0.937 | 1,12 | 0.007 | 0.934 |
|  | GT-Gi | | | | | | | | |
|  | Lever contacts | | | Probability lever | | | Latency lever | | |
|  | DF | F | p | DF | F | p | DF | F | p |
| Treatment | 1,8 | 23.653 | **<0.01** | 1,8 | 22.304 | **<0.01** | 1,8 | 19.311 | **<0.05** |
| Session | 1,8 | 6.706 | **<0.05** | 1,8 | 10.769 | **<0.05** | 1,8 | 9.333 | **<0.05** |
| Treatment*Session | 1,8 | 3.144 | 0.114 | 1,8 | 3.276 | 0.108 | 1,8 | 2.777 | 0.134 |
