# Supplementary material for: The paraventricular thalamus is a critical mediator of top-down control of cue-motivated behavior in rats
Source: eLife. 2019 Sep 10;8:e49041. doi: 10.7554/eLife.49041 (PMC6739869; doi:10.7554/eLife.49041)
Supplement: Supplementary file 8. — The results of linear mixed model analyses are shown for the effect of treatment (VEH vs. CNO), session (3 vs. 6) and treatment x session interaction for magazine-directed behaviors (magazine entries, probability to enter the magazine and latency to enter the magazine). Analyses were conducted separately for each experimental group (ST-Gq, GT-Gi). Bolded values indicate statistical significance, p<0.05. [file elife-49041-supp8.docx]

**Supplementary file 8. Session 3 vs. Session 6 of PavCA training: magazine-directed behaviors.**

|  | Magazine-directed behaviors (Goal-tracking) | | | | | | | | |
| --- | --- | --- | --- | --- | --- | --- | --- | --- | --- |
|  | ST-Gq | | | | | | | | |
|  | Magazine entries | | | Probability magazine | | | Latency magazine | | |
|  | DF | F | p | DF | F | p | DF | F | p |
| Treatment | 1,12 | 0.558 | 0.470 | 1,12 | 0.306 | 0.590 | 1,12 | 0.617 | 0.448 |
| Session | 1,12 | 1.134 | 0.308 | 1,12 | 0.339 | 0.571 | 1,12 | 1.063 | 0.323 |
| Treatment*Session | 1,12 | 0.032 | 0.861 | 1,12 | 0.059 | 0.812 | 1,12 | 0.000 | 0.987 |
|  | GT-Gi | | | | | | | | |
|  | Magazine entries | | | Probability magazine | | | Latency magazine | | |
|  | DF | F | p | DF | F | p | DF | F | p |
| Treatment | 1,8 | 1.876 | 0.208 | 1,8 | 4.878 | 0.058 | 1,8 | 3.061 | 0.118 |
| Session | 1,8 | 1.178 | 0.309 | 1,8 | 1.385 | 0.273 | 1,8 | 0.441 | 0.525 |
| Treatment*Session | 1,8 | 13.131 | **<0.01** | 1,8 | 6.362 | **<0.05** | 1,8 | 7.785 | **<0.05** |
